# Implications of unconventional histological subtypes on magnetic resonance imaging and oncological outcomes in patients who have undergone radical prostatectomy

**DOI:** 10.1038/s41598-024-65681-2

**Published:** 2024-06-27

**Authors:** Koichiro Kurokawa, Yasutaka Yamada, Shinichi Sakamoto, Takuro Horikoshi, Kodai Sato, Sakie Nanba, Yoshihiro Kubota, Manato Kanesaka, Ayumi Fujimoto, Nobuyoshi Takeuchi, Hiroki Shibata, Tomokazu Sazuka, Yusuke Imamura, Toyonori Tsuzuki, Takashi Uno, Tomohiko Ichikawa

**Affiliations:** 1 Department of Urology, Chiba University Graduate School of Medicine, 1-8-1 Inohana, Chuo-Ku, Chiba-City, Chiba 260-8670 Japan; 2 Department of Radiology, Chiba University Graduate School of Medicine, Chiba, 2608677 Japan; 3 Department of Surgical Pathology, Aichi Medical University Hospital, Aichi, 4801195 Japan

**Keywords:** PI-RADS v2.1, Cribriform, Intra-ductal carcinoma of the prostate, Ductal carcinoma, Radical prostatectomy, Cancer, Oncology, Urology

## Abstract

The prognostic significance of unconventional histology (UH) subtypes including intraductal carcinoma of the prostate (IDC-P), ductal adenocarcinoma, and cribriform pattern has been investigated for prostate cancer (PCa). However, little is known about magnetic resonance imaging (MRI) features and the oncological impact of tumor localization in localized PCa with UH. Clinical data of 211 patients with acinar adenocarcinoma (conventional histology [CH]) and 82 patients with UH who underwent robotic-assisted radical prostatectomy (RARP) were reviewed. Patients with UH are more likely to be older and have higher Gleason grade group, higher Prostate Imaging-Reporting and Data System (PI-RADS) v2.1 score, and larger tumor volume (TV) than those with CH. Multivariate analysis identified the presence of UH as an independent prognostic factor for progression-free survival (PFS) (hazard ration (HR) 2.41, 95% confidence interval (CI) 0.22–0.79, *P* = 0.0073). No significant difference in PFS was seen regarding tumor localization (transition zone [TZ] or peripheral zone [PZ]) in patients with UH (*P* = 0.8949), whereas PZ cancer showed shorter PFS in patients with CH (*P* = 0.0174). PCa with UH was associated with higher progression than PCa with CH among resection margin (RM)-negative cases (*P* < 0.0001). Further, increased PI-RADS v2.1 score did not correlate with larger TV in UH (*P* = 0.991), whereas a significant difference in TV was observed in CH (*P* < 0.0001). The prognostic significance of UH tumor was independent of tumor localization, and shorter PFS was observed even in RM-negative cases, indicating an aggressive subtype with micro-metastatic potential. Furthermore, UH tumors are more likely to harbor a large TV despite PI-RADS v2.1 score ≤ 3. These findings will help optimal perioperative management for PCa with UH.

## Introduction

Although significant advances have been accumulated in the treatment of advanced prostate cancer (PCa), this pathology remains the most commonly diagnosed cancer and second leading cause of cancer death among men in the United States^1^. The majority of cancer deaths are due to acquired resistance to treatment in metastatic PCa, and the prognosis for localized PCa is extremely favorable^2^. However, with the increasing understanding of epithelial histopathological subtypes, precise prognostic classification has become feasible, and unfavorable populations of localized PCa have been delineated^3^.

Acinar adenocarcinoma (AAC) is the major histological subtype of PCa^3^. The presence of subtypes with unconventional histology (UH) such as cribriform pattern, intraductal carcinoma of the prostate (IDC-P), and ductal adenocarcinoma has been implicated in adverse clinical outcomes as compared to conventional histology (CH)^3^. IDC-P was first demonstrated by Kovi et al. as a disease in which tumor cells penetrate into the prostatic ducts and acini as distinct from dysplasia^4,5^. Furthermore, McNeal et al. found that the presence of IDC-P was associated with significantly more advanced disease stage and increased risk of biochemical recurrence (BCR)^6^. Subsequent studies have identified UH as a factor associated with an adverse prognosis irrespective of disease stage and treatment^7–10^. The cribriform morphology of the prostate is defined as a confluent sheet of contiguous malignant epithelial cells with multiple, easily identifiable glandular lumens^11^. The cribriform component has been identified as an unfavorable prognostic factor following radical prostatectomy (RP), similar to IDC-P^12,13^. In addition, prostatic ductal adenocarcinoma has been considered a rare and aggressive histological subtype, accounting for 2.6% of PCa^11,14^. Notably, recent genomic analyses have revealed that these UH subtypes harbor a higher frequency of genetic alterations to tumor suppressor genes (e.g., loss of retinoblastoma 1 [*RB1*], tumor protein 53 [*TP53*], phosphatase and tensin homolog deleted from chromosome 10 [*PTEN*]), supporting the proposed clinical aggressiveness^15,16^. Thus, the growing understanding of UH subtypes implies the importance of early detection for these lesions.

With the introduction of the Prostate Imaging-Reporting and Data System (PI-RADS) and substantial advances in imaging technology, significant improvements in prostate biopsy diagnostic accuracy have been reported, and more information is available prior to surgery^17,18^. However, little is known about the correlation between magnetic resonance imaging (MRI) findings (including PI-RADS v2.1 score) and UH subtypes. The present study focused on tumor localization and its prognostic significance in patients with UH.

Herein, we explored the implications of the presence of UH subtypes on MRI and oncological outcomes after RP. Our findings will help with decision-making for the pre- and post-operative management of localized PCa in patients with UH.

## Results

### Background characteristics of patients

Among the 293 patients, 211 patients were diagnosed with AAC (CH) and 82 patients were diagnosed with UH. 9.6% of patients underwent MRI-fusion targeted trans-perineal prostate biopsy, while the others underwent only systematic biopsy. The median number of biopsy cores was 8 (6–26). UH were observed in 11 cases (13.4%) at the time of biopsy. Clinical data are summarized in Table 1. Patients with UH were more likely to be older (*P* = 0.0027) and have higher bGG (*P* < 0.001), higher PI-RADS v2.1 score (*P* = 0.0443), higher pGG (*P* = 0.0056), and higher TV (*P* = 0.0002) as compared to patients with CH. No significant difference in initial PSA level was seen (7.31 ng/mL in CH, 7.96 ng/mL in UH; *P* = 0.6621). 50.5% of our study participant received lymph node dissection and the frequency in patients with UH were significantly higher than those with CH (98.8% vs 31.8%, *P* < 0.001). Our study included approximately 90% intermediate- and high-risk patients using the national comprehensive cancer network (NCCN) and European Association of Urology (EAU) guidelines. This rate is higher than previously reported^19^ and may have resulted in a relatively high incidence of CH. Median observation period was 43 months in this study.

**Table 1:** Patients' characteristics by histological types.

	Pathological type		Total (*n* = 293)	*P* value
	With CH (*n* = 211 )	With UH (*n* = 82)		
Median age (range), years	67 (46–77)	70 (46–77)	67 (46–77)	0.0027^#^
Median initial PSA (range), ng/mL	7.31 (2.3–87.16)	7.96 (0.603–75.27)	7.6 (0.603–87.16)	0.6621^#^
Biopsy positive core, % (range)	27 (6–100)	38 (6–100)	27 (6–100)	0.1181^#^
Biopsy GG, n (%)				< 0.001*
≤ 3	170 (80.6)	44 (53.7)	214 (73)	
4	24 (11.4)	28 (34.1)	51 (17.4)	
5	17 (8.0)	10 (12.2)	28 (9.6)	
Pathological types				–
Acinar adenocarcinoma	211 (100)	0 (0)		
IDC-P	0 (0)	3 (3.7)		
Ductal	0 (0)	14 (17.1)		
Cribriform	0 (0)	67 (81.7)		
Mixed	0 (0)	5 (6.1)		
cT stage, n (%)				0.2636*
≤ 2c	192(91.0)	71 (86.6)	263 (89.8)	
≥ 3a	19 (9.0)	11 (13.4)	30 (10.2)	
cN stage, n (%)				
0	211 (100)	82 (100)		
1	0	0		
NCCN risk classification				< 0.001*
Low	32 (15.2)	2 (2.4)	34 (11.6)	
Intermediate	123 (58.3)	39 (47.6)	162 (55.3)	
High	56 (26.5)	41 (50)	97 (33.1)	
EAU risk classification				< 0.001*
Low	32 (15.2)	2 (2.4)	34 (11.6)	
Intermediate	114 (54)	32 (39)	146 (49.8)	
High	65 (30.8)	48 (58.5)	113 (38.6)	
Location of MRI, n (%)				0.0706*
TZ	79 (37.4)	22 (26.8)	101 (34.5)	
PZ	132 (62.6)	59 (72.0)	191 (65.2)	
PI-RADS v2.1 score, n (%)				0.0443*
≤ 3	63 (29.9)	14 (17.1)	77 (26.3)	
≥ 4	148 (70.1)	67 (81.7)	215 (73.4)	
pT stage, n (%)				0.0794*
≤ 2c	158 (74.9)	53 (64.6)	211 (72)	
≥ 3a	53 (25.1)	29 (35.4)	82 (28)	
Lymph node dissection				< 0.001*
Yes	67 (31.8)	81 (98.8)	148 (50.5)	
No	144 (68.2)	1 (1.22)	145 (49.5)	
pN stage, n (%)				0.3234*
Positive	2 (0.9)	2 (2.4)	4(1.4)	
Pathological GG, n (%)				0.0056*
≤ 3	169 (80.1)	53 (64.7)	222 (75.8)	
4	16 (7.6)	17 (20.7)	33 (11.2)	
5	26 (12.3)	12 (14.6)	38 (13)	
Median tumor volume, (range), cm^3^	2.13 (0.03–25.96)	2.89 (0.18–37.63)	2.29 (0.03–37.63)	0.0002^#^
RM positive, n (%)	63 (29.9)	25 (30.5)	88 (30)	0.9159*
Disease progression, n (%)	32 (15.2)	24 (29.3)	57 (19.5)	0.0062*

*CH* conventional histology, *UH* unconventional histology, *PSA* prostate-specific antigen, *GG* gleason grade group, *IDC-P* intra-ductal carcinoma of the prostate, *NCCN* National Comprehenesive Cancer Network, *EAU* European Association of Urology, *PI-RADS* prostate imaging reporting and data system, *pT* pathological T, *pN* pathological N, *RM* resection margin.

^#^Student's t-test.

*χ^2^ test.

### Clinical impact of the presence of UH in patients after RP

**Figure 1:**
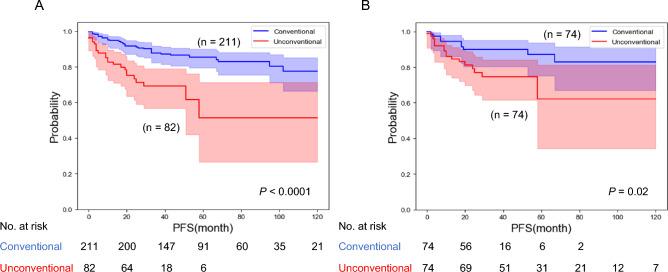
(**A**) Kaplan–Meier analysis by histological subtype (UH vs CH) for progression-free survival (PFS). (**B**) Kaplan–Meier analysis by histological subtype (UH vs CH) for PFS after propensity score-matching.

Patients with UH showed shorter PFS as compared to patients with CH in Kaplan–Meier analysis (*P* < 0.0001) (Fig. 1A). Three-year PFS was 68.8% with UH and 87.8% with CH. To mitigate differences in background, we performed propensity score-matching (PSM) analysis. After 1:1 PSM based on age, initial PSA, bGG, clinical T stage, and PI-RADS v2.1 score, 148 patients (74 in each group) were selected. Patients’ characteristics after PSM were shown in Supplementary Table 1. UH tumors were associated with worse outcomes when compared with CH tumors (*P* = 0.02, Fig. 1B).

Cox proportional hazard models was used to validate the prognostic impact of clinical parameters, including UH. After multivariate analysis, bGG (≥ 4) (hazard ratio [HR] = 3.02,* P* = 0.0002), clinical T stage (≥ 3a) (HR 3.13, *P* = 0.0007), presence of UH (HR 2.31, *P* = 0.005), TV (> 2.29 mL) (HR 2.7, *P* = 0.0113), and positive RM (HR 2.42, *P* = 0.0027) were identified as independent prognostic factors for PFS (Table 2).

**Table 2:** Uni- and multivariate cox proportional hazard models for PFS.

	Univariate			Multivariate		
	HR	95% CI	*P* value	HR	95% CI	*P* value
Age (> 67)	1.23	0.72–2.08	0.4533	–	–	–
Initial PSA (> 7.6)	1.54	0.90–2.65	0.1117	–	–	–
Biopsy positive core % (> 27)	2.29	1.29–4.05	0.0032	1.11	0.59–2.10	0.7495
Biopsy GG (≥ 4)	4.93	2.88–8.45	< 0.0001	3.02	1.68–5.43	0.0002
cT (3a)	5.9	3.25–10.72	< 0.0001	3.13	1.62–6.04	0.0007
PI-RADS v2.1 score (≥ 4)	2.36	1.11–4.99	0.0137	1.04	0.48–2.28	0.9178
Presence of UH	3.26	1.87–5.69	< 0.0001	2.31	1.29–4.15	0.005
pT (≥ 3a)	3.95	2.31–6.73	< 0.0001	–	–	–
pN	4.16	1.01–17.09	0.106	1.56	0.36–6.72	0.5502
Pathological GG (≥ 4)	5.03	2.94–8.60	< 0.0001	–	–	–
Tumor volume (> 2.29 mL)	4.23	2.31–8.4	< 0.0001	2.7	1.25–5.81	0.0113
RM	3.27	1.91–5.67	< 0.0001	2.43	1.36–4.33	0.0027

*PFS* progression-free survival, *PSA* prostate-specific antigen, *GG* gleason grade group, *PI-RADS* prostate imaging reporting and data system, *pT* pathological T, *pN* pathological N, *RM* resection margin.

### Prognostic significance of tumor localization and RM status in UH cancer

**Figure 2:**
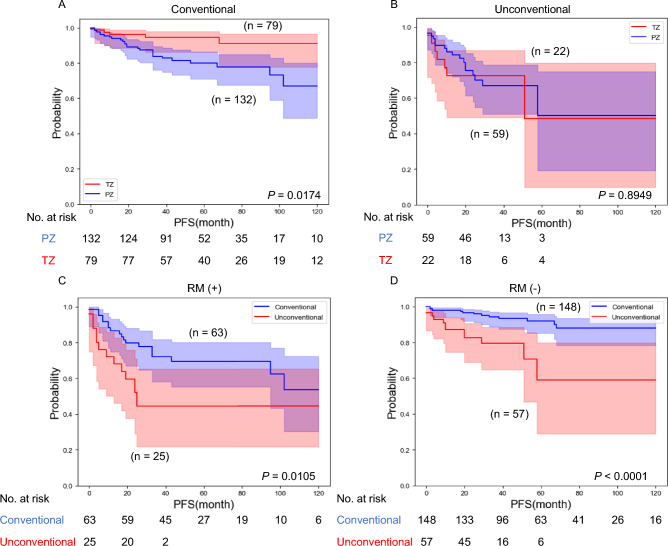
(**A**) Kaplan–Meier analysis by tumor localization (TZ vs PZ) for progression-free survival (PFS) in patients with CH. (**B**) Kaplan–Meier analysis by tumor localization (TZ vs PZ) for PFS in patients with UH. (**C**) Kaplan–Meier analysis by histological subtype (UH vs CH) for PFS in resection margin (RM)-positive cases. (**D**) Kaplan–Meier analysis by histological subtype (UH vs CH) for PFS in RM-negative cases.

We explored the prognostic impact of tumor localization (TZ or peripheral zone [PZ]). Of note, no significant difference in PFS was observed between TZ and PZ tumors in patients with UH (*P* = 0.8949), whereas PZ tumor was associated with increased risk of disease progression compared to TZ tumor in patients with CH (*P* = 0.0174) (Fig. 2A,B).

We then examined the impact of histological type on progression regarding surgical resection margin (RM) status. Interestingly, presence of UH tumor correlated strongly with unfavorable outcome in both RM-negative cases (*P* < 0.0001) and RM-positive cases (*P* = 0.0105) (Fig. 2C,D). Tumor localization has been considered to be related to RM status and our study showed that PZ tumors were more likely to be RM-positive (34.7%) than TZ tumors (21%, *P* = 0.0134; Fig. S1). These findings showed that UH tumor was associated with unfavorable oncological outcomes irrespective of tumor localization and curative resection.

### Correlation between PI-RADs v2.1 score and TV in UH cancer

**Figure 3:**
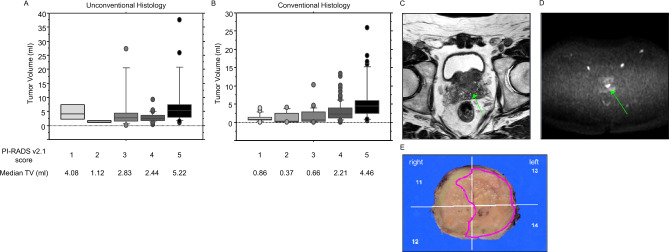
(**A**) Tumor volume classified by PI-RADS v2.1 scores in patients with UH. (**B**) Tumor volume classified by PI-RADS v2.1 scores in patients with CH. C) Prostate MRI (T2WI) of a representative case. D) Prostate MRI (DWI) of a representative case. E) Image of the prostatectomy specimen from a representative case.

We further investigated relationships between preoperative PI-RADS v2.1 score and TV as calculated from prostatectomy specimens. We expected preoperative PI-RADS v2.1 score and TV to show a positive relationship, since the PI-RADS scoring system was originally proposed to represent cancer lesions and aggressiveness^18^. Intriguingly, a positive correlation was observed in CH tumors while not in UH tumors (Fig. 3A,B). We examined the percentages of TV greater than 3.5 mL (approximately a 1.5 cm cube) in patients with PI-RADS ≤ 3 and found that UH tumors had 42.9% (6/14 cases) while CH had 12.7% (8/63 cases) (*P* = 0.0148), indicating that UH tumors are more likely to have a larger TV despite equivocal in MRI findings. A representative patient with ductal carcinoma, PI-RADS v2.1 score 3 and a large lesion (27.45 mL) occupying the left lobe showed a discrepancy between radiographic findings and tumor burden (Fig. 3C–E). Furthermore, we found that patients with UH were more likely to had upstage from ≥ cT2 to pT3 ≤ than those with CH (23.17 vs 19.9%). These finding indicated that UH tumors may be more difficult to detect on MRI as compared to CH tumors.

## Discussion

Our study demonstrated that the presence of a UH subtype correlated with increased risk of progression as compared to CH PCa and represented an independent prognostic factor for progression following RP. A systematic review and meta-analysis reported that intraductal disease correlated with increased risk of BCR (HR 2.09) and cancer-specific death (HR 2.93) for localized PCa^10^. Furthermore, shorter overall survival was observed for intraductal disease in patients with advanced PCa (HR 1.75)^10^. Intraductal disease thus presents histopathological features of a biologically and clinically aggressive subtype, irrespective of disease stage^10^. Meanwhile, the incidence of IDC-P reportedly varies depending on tumor stage, and metastatic and castration-resistant PCa has a higher prevalence than localized and hormone-sensitive PCa^20^. The presence of UH has been considered to exhibit treatment resistance to intensity-modulated radiation therapy, androgen-deprivation therapy, and chemotherapy in addition to surgery^7,9,21^. Wei et al. indicated that surgical treatment was a favorable option in patients with ductal carcinoma of the prostate as compared to radiation and/or hormonal therapy after PSM analysis^22^. Further investigation is warranted to identify better management options for hard-to-treat histological subtypes.

In addition, the prognosis of UH tumor did not correlate with tumor localization (TZ or PZ), although a significant relationship was observed in CH tumors. The presence of UH was associated with poor PFS even in cases with negative surgical margins. Notably, one patient with UH and negative RM had postoperative metastases. These findings indicated an aggressive phenotype with micro-metastatic potential for UH cancer prior to local treatment. Our group has previously reported that radiological location in PZ tumor was associated with higher incidence of progression than that in TZ tumor^23,24^. The present data showed that PZ tumors are more likely to be RM-positive than TZ tumors, resulting in adverse outcomes. Based on these findings, UH tumors have the potential to represent a more aggressive phenotype and metastatic potential than CH tumors. Xu et al. found that IDC-P was a risk factor for pathological lymph node metastasis in patients with cT2N0M0 stage who underwent RP^25^. Comprehensive genome analysis revealed a higher frequency of alterations in tumor suppressor genes (e.g., *TP53*, *RB1*) for UH cancers, which may manifest as these clinical features^26^. Furthermore, *PTEN* deficiency was frequently observed in patients with IDC-P, at 70–90%^27^. A correlation between IDC-P and DNA damage repair gene alterations has been reported, and the National Comprehensive Cancer Network (NCCN) guidelines recommend genetic testing for patients with a family history of PCa if IDC-P is detected in prostate biopsy ^28^. Meanwhile, Ito et al. demonstrated a lower frequency of *PTEN* loss in Asian patients than in Western populations^29^. Thus, although evidence is accumulating regarding genomic alterations of PCa with UH, large-scale genomic analyses across ethnic groups is warranted to unravel the underlying molecular mechanisms.

Our study revealed that elevated PI-RADS v2.1 score did not correlate with larger TV in UH tumors, whereas a significant positive correlation was seen in CH tumors, indicating that UH tumor may diminish the ability to detecting cancerous lesions on preoperative MRI. Discrepancies between radiographic findings and prostatectomy specimens have been examined and previous reports have demonstrated that a significant number of clinically significant (cs)PCa could not be detected before surgery^30–32^. Those studies investigated predictors for the diagnosis of csPCa in MRI-negative cases. Clinical parameters including PSA density, family history, and prior biopsy results were identified as predictors for csPCa, but the impact of histological subtype has never been studied^30,31^. Similar to the present findings, a previous report revealed that 90.7% of patients with ductal adenocarcinoma and IDC-P showed PI-RADS score ≥ 4 on preoperative MRI^33^. On the other hand, our in-depth analysis indicated that UH tumors may have larger TV even in cases with PI-RADS scores of 3 or less, and that preoperative PI-RADS scores did not positively correlate with TV in surgical specimens. To the best of our knowledge, this study is the first to compare preoperative MRI and postoperative TV for UH PCa. Further research including elucidation of the molecular mechanisms is required to address this disparity.

This study showed several limitations that should be kept in mind. First, our analysis was conducted retrospectively. Second, the patient population was relatively small. Lastly, UH subtypes were combined and analyzed as a single group. Larger, prospective cohort studies with long-term follow-up are warranted to verify our findings. In addition, detailed analyses of each histological subtype are needed.

## Conclusions

This study demonstrated that the presence of UH was associated with a higher progression rate than CH-only tumors following RARP. Clinical aggressiveness was not dependent on tumor localization or treatment radicality, indicating a metastatic potential of UH subtypes. In addition, a discrepancy is more likely to arise between tumor quantity on preoperative MRI and in prostatectomy specimens for UH tumors. These findings will help unravel the clinical features of UH in PCa and facilitate the development of optimal perioperative management for PCa patients with UH.

## Methods

### Patient criteria

A total of 293 patients who underwent robotic-assisted RP (RARP) at Chiba University Hospital between 2016 and 2020 were included in this study. All patients received prostate needle biopsies and were diagnosed with PCa. Computed tomography (CT), and ^99m^technetium‐methylene‐diphosphate (^99m^Tc‐MDP) bone scintigraphy were used to detect metastatic lesions prior to surgery. RARP was performed without neoadjuvant hormone therapy. Prostatectomy specimens were all evaluated by the pathologist and diagnosed with AAC or UH subtypes including IDC-P, ductal adenocarcinoma, or cribriform pattern. No adjuvant radiotherapy (RT) was performed and only salvage therapy was considered in our study. This study was approved by the Clinical Research Ethics Review Committee of Chiba University Hospital and informed consent was obtained from all patients. Our study was conducted in accordance with ethical standards that promote and ensure respect and integrity for all human subjects and the Declaration of Helsinki. All experiments in the present study were performed in accordance with relevant named guidelines and regulations.

### Clinical parameters and oncological outcomes

We obtained the following clinical parameters for each patient: age at operation; initial prostate-specific antigen (PSA) level; percentage of positive biopsy cores; biopsy Gleason grade group (bGG); clinical TNM classification; preoperative PI-RADS version 2.1 score; and pathological findings from the prostatectomy specimen.

The following method was used to measure tumor volumes (TVs) from prostatectomy specimens^34^. All specimens were sectioned transversely at 5-mm intervals and submitted as whole sections. If multiple tumors were present, only the index tumor was measured. All slides containing cancer lesions were imported into ImageJ software (National Institutes of Health). Tumor volume was determined by scanning the specimen sections and analyzing the area of the tumor using ImageJ. The following formula was used: total tumor volume (mL) = tumor area × specimen thickness × 1.1 (corrected for shrinkage)^34^.

Prostate Cancer Clinical Trial Working Group 2 (PCWG2) criteria were used to define disease progression in this study^35^. Progression was determined as a PSA concentration ≥ 0.2 ng/mL following RARP, measured on two consecutive occasions with an interval of at least 2 weeks. The date of surgery was defined as the date of progression if PSA level did not reach ≥ 0.2 ng/mL postoperatively.

### PI-RADS v2.1 scoring system

All patients underwent 3-T MRI of the prostate at prior to prostate biopsy. MRI was performed using T1-weighted, T2-weighted, and diffusion-weighted imaging (DWI) sequences to produce an apparent diffusion coefficient map. A high b value (b = 2000) was used for DWI. MRI consisted of T2-weighted imaging and DWI. Both bi-parametric MRI (bp-MRI) comprising T2-weighted imaging and DWI, and the apparent diffusion coefficient map were employed by the radiologist to determine the PI-RADS v2.1 score.

PI-RADS v2.1 scores were assessed by the radiologist with non-contrast bp-MRI. The score for each patient was documented using the PI-RADS v2.1 method (5-point scale). Modifications implemented in PI-RADS v2.1 were scoring of DWI in all zones in categories 2–3 and revised scoring of the overall rating category in transition zones (TZs). A DWI score of 4 or 5 elevated the overall PI-RADS rating category from 2 to 3 for lesions with a T2W score of 2 in a TZ^36^.

### Statistical analysis

Student’s t-test and the *χ*^2^ test were used for comparisons between groups. Kaplan–Meier methods and Cox proportional hazard models were used to validate outcomes and predictive factors. Multivariate analysis was performed using those clinical parameters showing statistical significance in univariate analyses. JMP Pro 15 software (SAS Institute, Tokyo, Japan) was implemented for statistical analyses. Values of *P* < 0.05 were considered statistically significant in this study.

### Supplementary Information

Supplementary Figure S1.

Supplementary Legends.

Supplementary Table S1.

## Data Availability

The data presented in this study are available on request to the corresponding author.
