# Supplementary figures and images for: Implications of unconventional histological subtypes on magnetic resonance imaging and oncological outcomes in patients who have undergone radical prostatectomy
Source: Sci Rep. 2024 Jun 27;14:14868. doi: 10.1038/s41598-024-65681-2 (PMC11211384; doi:10.1038/s41598-024-65681-2)

## Slide 1
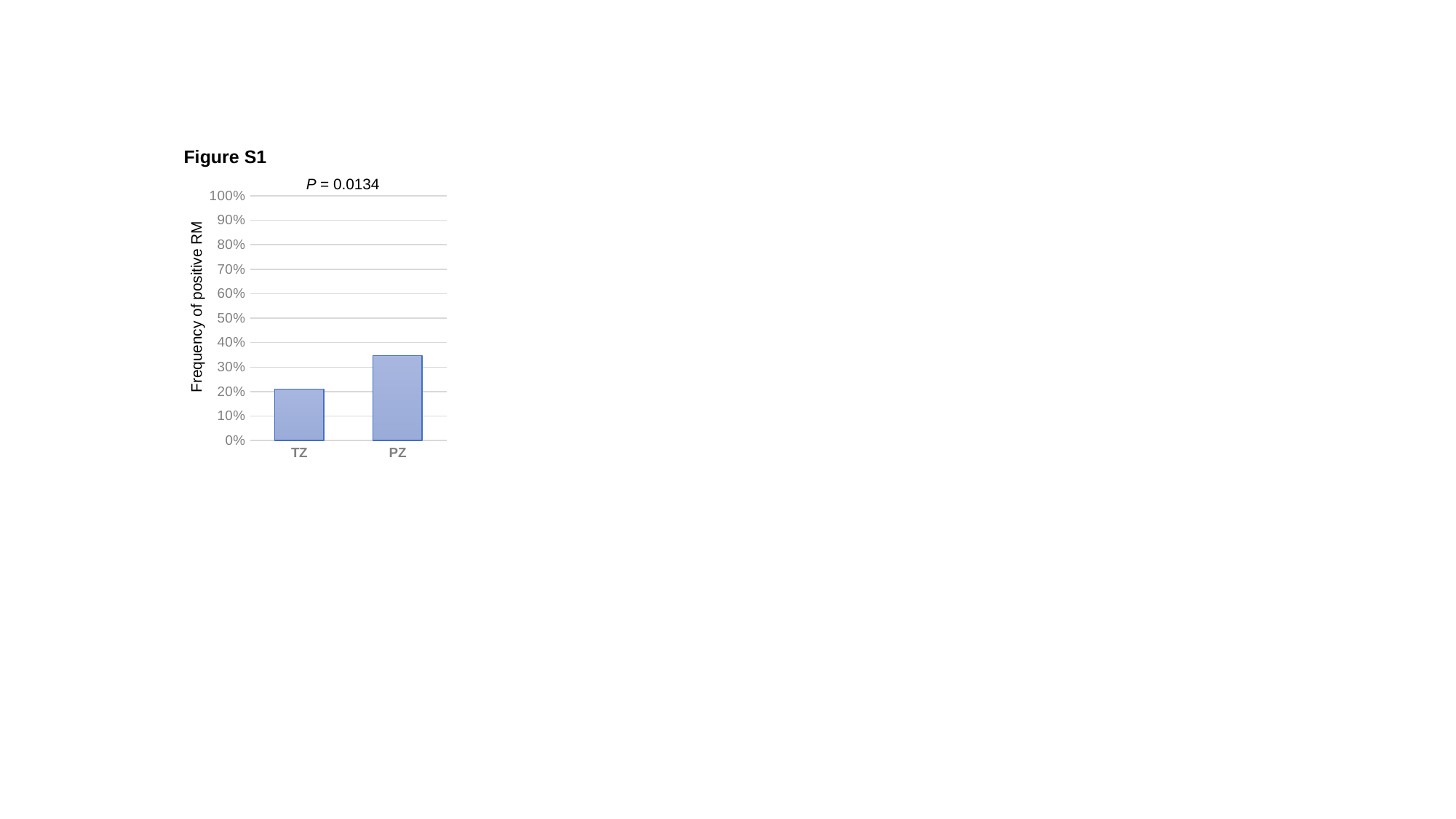

Figure S1
P = 0.0134
### Chart
| Category | |
|---|---|
| TZ | 0.21 |
| PZ | 0.347 |Frequency of positive RM

Supplement: Supplementary file 1 — Supplementary Figure S1. [file 41598_2024_65681_MOESM1_ESM.pptx]
