# Supplementary material for: Implications of unconventional histological subtypes on magnetic resonance imaging and oncological outcomes in patients who have undergone radical prostatectomy
Source: Sci Rep. 2024 Jun 27;14:14868. doi: 10.1038/s41598-024-65681-2 (PMC11211384; doi:10.1038/s41598-024-65681-2)
Supplement: Supplementary file 2 — Supplementary Legends. [file 41598_2024_65681_MOESM2_ESM.docx]

**Supporting Information**

Figure S1. Frequency of positive resection margin (RM) in TZ and PZ tumors.

Table S1. Patients' characteristics after propensity score-matching.
